# Toll-like receptor 4 signaling activation domains promote CAR T cell function against solid tumors

**DOI:** 10.1016/j.omton.2024.200815

**Published:** 2024-05-14

**Authors:** Veronika Mikolič, Jelica Pantović-Žalig, Špela Malenšek, Matjaž Sever, Duško Lainšček, Roman Jerala

**Affiliations:** 1 Department of Hematology, University Medical Centre Ljubljana, 1000 Ljubljana, Slovenia; 2 Graduate School of Biomedicine, University of Ljubljana, 1000 Ljubljana, Slovenia; 3 Department of Synthetic Biology and Immunology, National Institute of Chemistry, 1000 Ljubljana, Slovenia; 4 Faculty of Medicine, University of Ljubljana, 1000 Ljubljana, Slovenia; 5 Centre for Technologies of Gene and Cell Therapy, National Institute of Chemistry, 1000 Ljubljana, Slovenia

**Keywords:** CAR T cell therapy, cancer immunotherapy, innate immunity, TLR, costimulation

## Abstract

Chimeric antigen receptor (CAR) T cell therapy has emerged as a powerful therapeutic approach against a range of hematologic malignancies. While the incorporation of CD28 or 4-1BB costimulatory signaling domains into CARs revolutionized immune responses, there is an exciting prospect of further enhancing CAR functionality. Here, we investigated the design of CD19 CARs enriched with distinct Toll-like receptor 4 (TLR4), myeloid differentiation primary response 88 (MyD88), or Toll/IL-1 domain-containing adaptor-inducing interferon (IFN)-β (TRIF) costimulatory domains. Screening of various designs identified several candidates with no tonic activity but with increased CD19 target cell-dependent interleukin (IL)-2 production. Human T cells transduced with the selected CAR construct exhibited augmented hIL-2 and hIFN-γ induction and cytotoxicity when cocultured with CD19-positive lymphoma and solid-tumor cell lines. RNA sequencing (RNA-seq) analysis demonstrated the upregulation of some genes involved in the innate immune response and T cell activation and proliferation. In experiments on a xenogeneic solid-tumor mice model, MyD88 and TLR4 CAR T cells exhibited prolonged remission. This study demonstrates that the integration of a truncated TLR4 signaling costimulatory domain could provide immunotherapeutic potential against both hematologic malignancies and solid tumors.

## Graphical abstract

**Figure:**
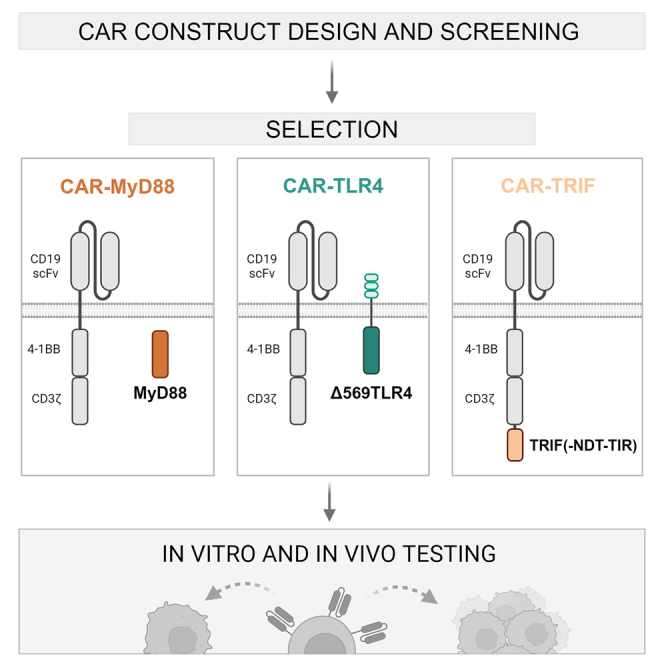

## Teaser

Lainšček and colleagues present novel chimeric antigen receptor (CAR) T cell designs that incorporate Toll-like receptor 4 (TLR4) activation domains. They observed enhanced cytokine production and target cell killing *in vitro*, and experiments in solid-tumor mouse models exhibited prolonged remission, holding promise for improved CAR T cell treatments.

## Introduction

Over the past decade, chimeric antigen receptor (CAR) T cell therapy has emerged as an effective immunotherapeutic therapy against hematologic malignancies, and several CAR T products have already been approved for clinical use.^1^ As CAR T cells showed great therapeutic features in hematologic cancers, the need to treat solid tumors also arose. In addition to inadequate specific antigen targets or their poor accessibility, the therapeutic potential of CAR T cell therapy in solid tumors is further hindered not only by insufficient tumor infiltration of therapeutic CAR T cells but also by complex interactions between tumor cells and their surrounding tumor microenvironment (TME),^2^^,^^3^ leading to metabolic starvation of CAR T cells. Additionally, the therapeutic outcomes of CAR T cell-based immunotherapies may be limited by transient persistence and inadequate proliferation of CAR T cells within the host.^4^ Consequently, there is a need for studies aiming to enhance CAR T cell functionality, thereby overcoming TME challenges and optimizing CAR T cell therapy efficacy. Intracellular signaling domains of genetically modified CARs currently used in clinical settings originate from T cell receptors and their costimulatory domains.^5^^,^^6^ In addition to the conventional signaling molecules, various signaling domains and bioengineering approaches are used to improve the safety and efficacy of treatments.^1^ A range of other costimulatory domains, including elements of innate immunity for boosting CAR T cell efficacy, were assessed.^7^ Toll-like receptors (TLRs) are predominantly expressed by innate immune cells. However, TLRs are also important costimulatory and regulatory molecules within T cells.^8^^,^^9^^,^^10^ By directly stimulating T cells, TLR agonists can enhance cytokine production, increase T cell sensitivity to stimulation, promote T cell memory, and reduce the activity of regulatory T cells.^11^^,^^12^^,^^13^^,^^14^^,^^15^ TLRs transmit signals through Toll/interleukin (IL)-1 receptor (TIR) domains, which interact with cytoplasmic adaptor proteins, including myeloid differentiation primary response 88 (MyD88), MyD88 adapter-like (MAL)/TIRAP, TIR domain-containing adaptor-inducing interferon (IFN)-β (TRIF), and TRIF-related adapter molecule (TRAM). MyD88-dependent signaling is responsible for the production of proinflammatory cytokines and chemokines, while activation through TRIF initiates signaling pathways resulting in the production of type I IFNs.^16^^,^^17^^,^^18^ Incorporation of TLR signaling domains into CAR T cells may represent a promising strategy to exploit T cell-intrinsic TLR function. This idea was already tested in the form of CAR with a CD28 costimulatory domain in combination with MyD88 and CD40,^19^^,^^20^^,^^21^ where the constructs exhibited substantial tonic activity. Additionally, another design incorporated a combination of CD28 and the TLR2 TIR domain.^22^^,^^23^ Here, we developed anti-CD19 CARs that combine the TNF-receptor superfamily member 4-1BB (CD137, TNFRSF9) costimulatory domain with TLR4 signaling activation domains. We included not only additional MyD88 protein-based constructs but also other TLR4 signaling domains that were, to our knowledge, not tested previously. Incorporation of domains of TRIF and TLR4 into CARs showed promising results, presenting new designs of CAR constructs that may be beneficial in treating solid tumors, as prolonged CAR T cell persistence of CAR TLR4 was observed compared to conventional CAR T cells, demonstrating TLR4 domain as the best candidate for increasing CAR T cell potency.

## Results

### TLR4 signaling costimulatory domain enhances activation in the T cell line

**Figure 1:**
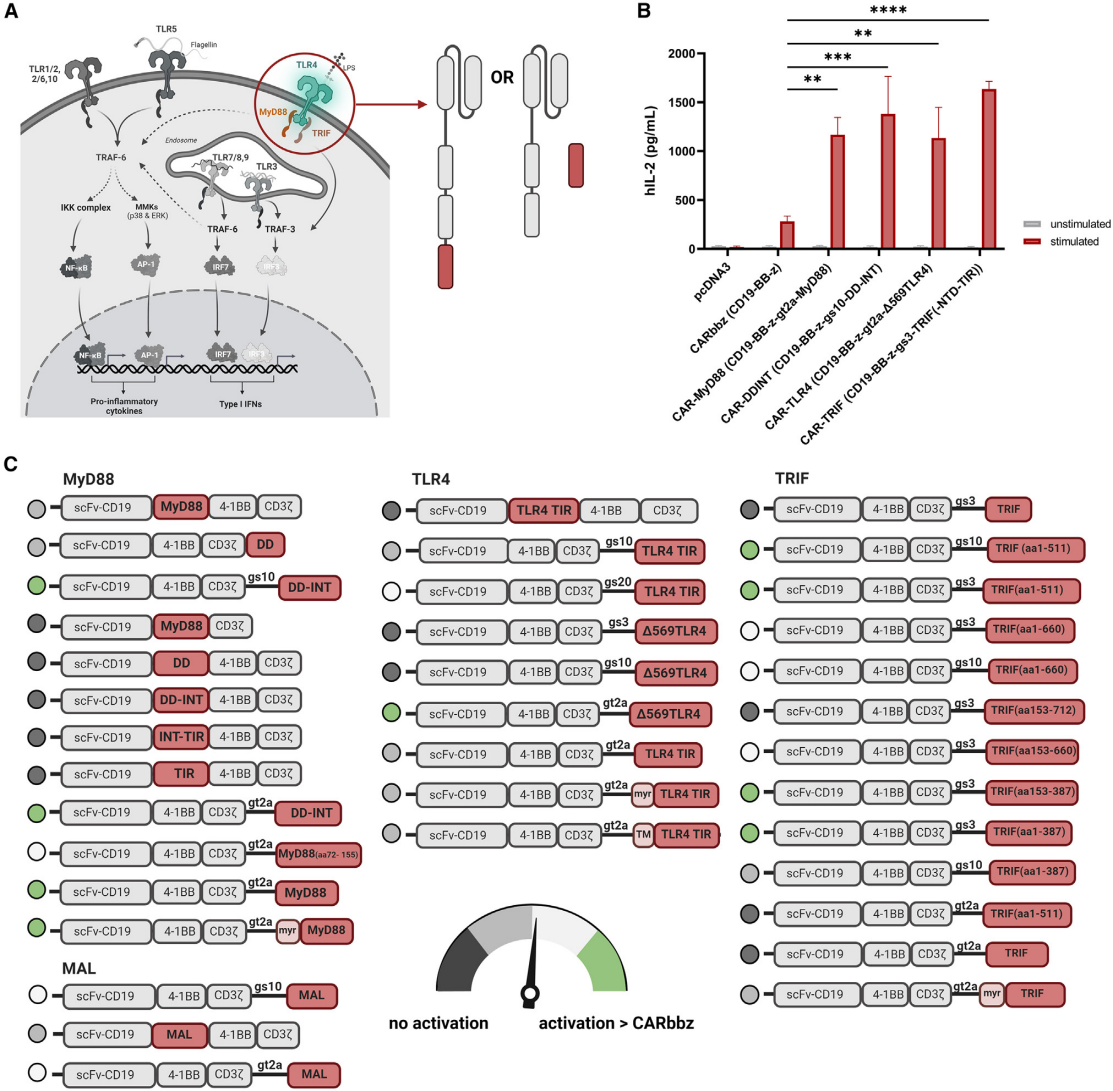
Determination of TLR4 signaling costimulatory domains for potentiation of CAR activity (A) A visual representation illustrates the TLR4 signaling-derived costimulatory domain selected for integration in CARs, along with canonical TLR signaling pathways that can be transduced using these CARs. (B) Jurkat cells were electroporated with pDNA of selected constructs. The next day, 1.5 × 10^5^ electroporated cells were cocultured with target CD19-positive Raji cells at an E:T ratio of 10:1. IL-2 levels were quantified after 48 h by ELISA. Data represent independent experiments (*n* = 4). The subsequent experiment refers to the selected constructs to their shortened name, while the entire construct name is indicated within round brackets in this figure. (C) Schematic representation of all tested plasmid CAR constructs and their activities. The activation indicator is based on the levels of IL-2 secretion in comparison to CARbbz alone. Figure S1F provides data for the results depicted in this illustration. Data are presented as the mean ± SEM values. Statistical differences were calculated by two-way ANOVA with Tukey’s multiple comparisons. ∗∗*p* < 0.01, ∗∗∗*p* < 0.001, ∗∗∗∗*p* < 0.0001.

We examined the effect of TLR4-derived costimulatory domains and their effect on CAR T cell immunotherapy, aiming to increase the potency and functionality of CD19 CAR T cells. To do so, we utilized a CD19-specific second-generation CAR with a CD8 transmembrane domain, 4-1BB costimulatory domain, and CD3ζ signaling domain as a benchmark (CARbbz) with protein partners derived from TLR4 signaling pathways. First, we focused on the key adaptor molecule, MyD88, as its effect has been to some extent already demonstrated by Foster et al.,^20^^,^^21^ where MyD88 lacking the TIR domain was used in combination with CD40. We sought to explore the effect of the coexpression of CAR and the activation domains of proteins involved in TLR4 signaling pathways on T cell activation. Jurkat cells, a human T cell line, were electroporated with CARbbz plasmid and MyD88-, TRIF-, TLR4-, or MAL domain-expressing plasmids. IL-2 secretion was measured after stimulation with target CD19+ Raji cells. Whole-length MyD88 and CD19 CARbbz coexpression significantly increased IL-2 production (Figure S1A) compared to CARbbz alone. Importantly, cells subjected to electroporation with the MyD88 plasmid alone did not produce IL-2, indicating the absence of constitutive activity resulting from MyD88 domain overexpression. Moreover, no IL-2 was detected following electroporation with a plasmid for myristoylated MyD88 (myrMyD88), a form resulting in the localization of MyD88 to the cell membrane^24^^,^^25^ (Figure S1A). We surmised that this modification could potentially facilitate Myddosomal complex formation, given that the cell membrane serves as the primary site for complex assembly.^26^ Next, we also conducted experiments in which Jurkat cells were electroporated with CARbbz and either TRIF-, TLR4-, TLR4 dimer- TIR-, or MAL-expressing plasmids, but, to our surprise, we did not observe significantly higher secretion of IL-2 cytokine, one of the key markers for T cell activation, compared to CARbbz alone when cocultivated with Raji cells (Figures S1B and S1C). It appears that TRIF not only reduces the capability of activation but also decreases cell viability, as we used construct, expressing whole TRIF protein, containing receptor-interacting protein homotypic interaction motif (RHIM) domain, which can cause apoptosis (Figure S1D).^27^ Based on these results, we designed and tested constructs containing additional TLR4 signaling-derived costimulatory domains. In the framework of our construct design, we decided to incorporate the whole signaling protein as well as specific structured elements associated with TLR4 activation (Figure S1E).^17^^,^^28^ Our initial CAR design idea, revolving around the canonical TLR signaling pathway, is presented in Figure 1A. To enable the detection of CAR expression, a Myc tag was introduced into all CAR constructs, allowing recognition by the anti-Myc antibody. We tested all constructs based on electroporation-mediated plasmid delivery into Jurkat cells with subsequent stimulation with the human CD19-positive Raji cell line, a cell model for B cell lymphoma. Constructs that generated higher activation based on IL-2 secretion compared to the second-generation CAR were selected for further experiments (Figure 1B). Among the several MyD88 variants we assessed, we selected the construct with full-length MyD88 as an additional costimulatory domain separated by the gt2a sequence and a construct comprising MyD88 lacking the TIR domain linked with a glycine-serine (gs)10 amino acid (aa) linker that genetically fuses CAR with the death domain (DD) of MyD88. We previously demonstrated that the N-terminal region of MyD88 is essential for efficient signaling via TLRs due to its interaction with the membrane.^24^ Similarly, in our designs, we observed that the CAR containing only residues 72–155 of MyD88 was less active than the constructs containing the complete DD and intermediate domain (INT) (1–155 aa) of MyD88 (Figure S1F), corroborating the importance of the N-terminal segment of MyD88 in potentiating the response. Previous studies suggested that covalent fusion with MyD88, as also tested here, resulted in the instability of CAR at the plasma membrane.^20^ Although we did not observe this effect in lentivirally transduced Jurkat cells (Figure S2A), this finding was confirmed later when transducing primary T cells with CAR-gs10-DDINT lentiviral vectors (Figure S2B). Consequently, our further experiments focused on a CAR construct with MyD88 incorporation via self-cleavage peptide (gt2a), rendering two separate functional polypeptide chains from a single mRNA transcript.^29^ From the pool of CARs with a TLR4 signaling costimulatory domain, we selected a construct composed of the N-terminal truncation of the ectodomain of human TLR4 (Δ569TLR4), separated by the gt2a peptide, which also resulted in two separate protein chains inserted into the plasma membrane. We previously showed that this truncated version exhibited constitutive activity by overexpression in HEK293 cells^30^; however, we did not observe constitutive activation in T cells (Figure 1B). Next, when designing CARs with a TRIF costimulatory domain, we wanted to avoid TRIF-induced apoptosis.^27^ As demonstrated in previous studies,^27^^,^^31^^,^^32^ a C-terminal segment comprising a RHIM domain and a C-terminal part with a TIR domain is sufficient to induce apoptosis; therefore, we designed a CAR construct with a TRIF-based costimulatory domain lacking an RHIM domain and constructs comprising the N-terminal 511 aa. We also assessed the constructs that lacked some other domains of TRIF. A construct incorporating a costimulatory TRIF domain (containing 153–387 aa, without N-terminal domain [NTD] and TIR domain), genetically fused with the gs3 linker, was chosen for further experiments, as we observed elevation in IL-2 secretion upon cocultivation of plasmid-electroporated Jurkat cells with Raji cells (Figure S1F). The remaining aa residues (153–387 aa) in the selected CAR-TRIF include the TANK-binding kinase 1 (TBK1) motif, TNF-receptor-associated factor (TRAF) 2, and TRAF6-binding motif involved in the activation of nuclear factor κB (NF-κB) and IFN regulatory factor 3 (IRF3).^33^^,^^34^^,^^35^ Evaluation of the CAR construct containing another TLR4 signaling mediator, MAL, in primary T cells was not pursued further, as we observed no significant increase in activation in any of the tested constructs based on this signaling mediator (Figures 1C and S1F).

### Increased CD19 CAR T cell activation following TLR4 signaling costimulation

**Figure 2:**
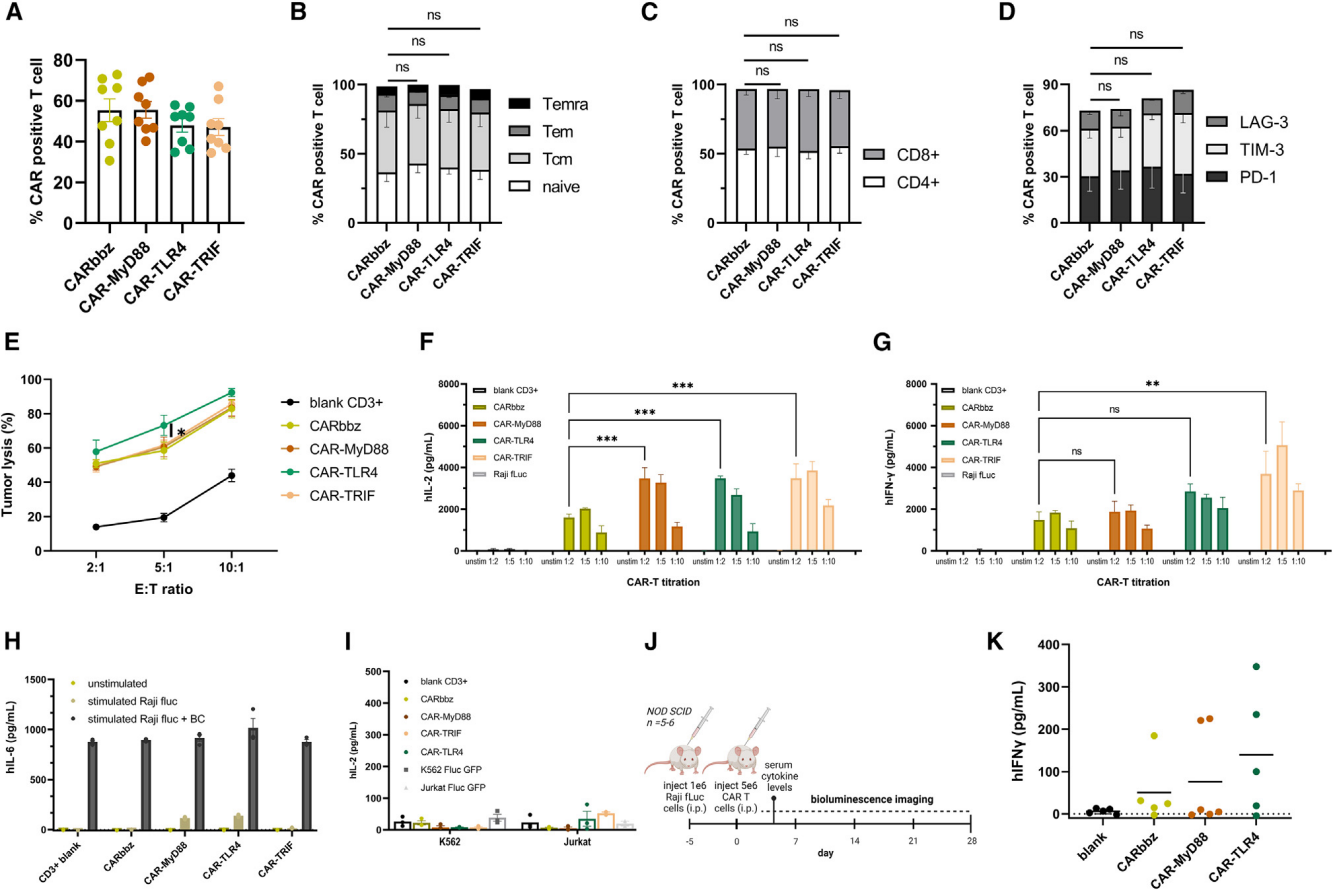
TLR4 signaling costimulatory domains enhance human CD19 CAR T cell function (A) The fraction of CD19 CAR-positive T cells measured between days 4 and 7 after lentiviral transduction in independent experiments within this study (*n* = 8). (B) CD19 CAR T cell subsets, classified into four differentiation subsets based on CD45RA and CD62L expression, measured by flow cytometry: naive (CD45RA^+^CD62L^+^), Tcm (CD45RA^−^CD62L^+^), Tem (CD45RA^−^CD62L^−^), and Temra (CD45RA^+^CD62L^−^). Assessment of all phenotype markers was performed after expansion and before functional assays. Data shown were collected from three different donors (*n* = 3). (C) Graph showing flow cytometry results for CD4^+^ and CD8^+^ CAR T cells. The presented data were collected from three different donors (*n* = 3). (D) Expression of LAG-3, TIM-3, and PD-1 exhaustion markers in different CD19 CAR T cell group populations before coculture with target CD19+ cells. Data showing results for two individual donors (*n* = 2). (E) CD19 CAR T cell cytotoxicity, determined *in vitro*. Luciferase-based killing assays were performed using Raji lymphoma-FLuc cell lines. The CD19 CAR T cells and target tumor cells were cocultured for 24 h at specified E:T ratios. Data were collected from two distinct donors, with three biological replicates conducted for each experiment (*n* = 2). Statistical significance is indicated between CARbbz and CAR-TLR4. (F) hIL-2 secretion and (G) hIFN-γ cytokine production following 48 h of coculture with target CD19+ cells at the indicated E:T ratios. Data were collected from two different donors with three biological replicates performed for each. (H) To analyze the effects of other immune cells, a coculture was prepared in three distinct conditions: unstimulated with media only, stimulated with Raji cells, and stimulated with both Raji cells and the addition of buffy coat (BC). The number of CAR T cells in all groups and conditions was 1.5 × 10^5^, with Raji cells comprising half the number of effector cells (E:T = 2:1). The presence of the cytokine hIL-6 was determined 48 h later by ELISA. (I) Levels of hIL-2 secretion were evaluated after 48 h in coculture with two different CD19-negative cell lines (K562, Jurkat), showing no constitutive activity. (J) A xenograft mouse lymphoma model was established by intraperitoneal injection of 1 × 10^6^ CD19+ Raji-FLuc cells. The indicated CAR T cells at a 5 × 10^6^ dose were intraperitoneally injected on day 5, followed by weekly bioluminescence imaging (n = 5–6). (K) hIFN-γ values in mice sera 3 days after CD19 CAR T cell treatment (n = 5–6). Each dot represents the value of an individual mouse. Data are the mean ± SEM. Statistical differences were calculated by two-way ANOVA with Tukey’s multiple comparisons. ∗*p* < 0.05, ∗∗*p* < 0.01, ∗∗∗*p* < 0.001.

While Jurkat cells can be useful for initial CAR screening,^36^ their functional response is incomplete compared to primary cells due to inherent dysfunctional pathways.^37^^,^^38^ To overcome this limitation, we transferred the most promising CARs, based on TLR4 signaling, into lentiviral vectors. We performed a more thorough evaluation of their functionality in primary human CD3+ cells that underwent lentiviral transduction. The efficiency of CAR T cell transduction was found to be comparable across all considered CAR constructs in our study, with a calculated mean of 51% (±5%) (Figure 2A), eliminating the possibility of the influence of differential expression. A comprehensive characterization using flow cytometry was conducted before functional analysis, which revealed no significant differences in T cell memory subpopulations (Figure 2B) or the ratio of CD4 to CD8 T cells among the various CAR T cell populations (Figure 2C). We also checked the expression of three exhaustion markers (TIM-3, LAG-3, and programmed cell death protein 1 [PD-1]) before conducting functional assays and found no significant differences (Figure 2D), although some higher PD-1 and TIM-3 values were observed in CAR-TLR4 cells. These observations suggest that the incorporation of an additional TLR-costimulatory domain does not result in statistically significant phenotypic alterations, nor does it have detrimental effects on CD19 CAR T cell fitness within the generated CAR T cells at the basal level. When we cocultured our CD19 CARs with two CD19-positive tumor cell lines, Raji-FLuc and BCWM-FLuc (Figures 2E and S2C), we observed comparable CD19 CAR-mediated cytotoxicity between CAR-MyD88, CAR-TRIF, and CARbbz, while CAR-TLR4 exhibited a statistically significant increase in the capacity to lyse target Raji cells. Additionally, we examined supernatants from these cocultures after 48 h and found an elevation in hIL-2 production and secretion for all TLR-costimulatory CARs, higher than the observed levels in CARbbz (Figure 2F), confirming improved CD19 CAR T functionality when costimulatory TLR signaling domains were introduced. In addition, we observed a significant increase in hIFN-γ in the CAR-TRIF group and a smaller increase in the CAR-TLR4 group (Figure 2G). We observed a small amount of hIL-6 in CAR-MyD88 and CAR-TLR4 supernatants upon stimulation with Raji cells, as depicted in Figure 2H. As we know that cytokine release syndrome (CRS) is mediated to a large degree by other immune cells, we assembled a more immunologically relevant microenvironment *in vitro*. We introduced additional immune cells in the form of a buffy coat to coculture CD19 CAR T cells and target Raji cells. This led to an increase in hIL-6 release, consistently observed in all evaluated cocultures, including control T cells (Figure 2H). These findings imply that the increased hIL-6 concentration levels would likely not be significantly higher in subjects treated with CAR MyD88 or CAR TLR4 *in vivo*. We also subjected our CAR T cells to CD19-negative cell lines (K562, Jurkat), and the results indicated an absence of nonspecific activation based on minimal cytokine production (Figure 2I), indicating that there is no substantial tonic signaling in the tested CARs.

Next, we sought to investigate whether enhanced CD19 CAR activity due to the incorporated TLR signaling domains results in increased cancer cell killing *in vivo*, therefore we established a NOD scid gamma (NSG) lymphoma mouse model. Lymphoma Raji-FLuc cells were introduced into the murine subjects with subsequent CD19 CAR T therapeutic cells (Figure 2J), and tumor growth was monitored by bioluminescence (BLI) imaging. (Figures S2D and S2E). Three days after the administration of CD19 CAR T cells, we measured cytokine levels in the serum samples. While hIL-2 was undetectable and mIL-6 values in treated mice were negligible (Figure S2F), we observed a moderate elevation in IFN-γ concentrations within the serum samples of mice treated with the TLR4 and MyD88 constructs in comparison to those in the CARbbz group (Figure 2K). The therapeutic effect was somewhat lower in mice subjected to treatment with CAR-MyD88 and CAR-TLR4 (Figures S2D and S2E). The response to CAR T cell therapy was observed within 2 weeks in the treated mice, with most mice displaying no evidence of the remaining tumor burden according to BLI. Nevertheless, one mouse in each CAR group was unresponsive to the treatment. Survival analysis in the mouse experiment revealed no significant difference between CAR groups (Figure S2G). Notably, none of the murine subjects experienced a loss exceeding 10% of their initial body mass throughout the duration of the experiment (Figure S2H), implicating no severe side effects of CAR T therapy based on body mass change and mouse IL-6 secretion.

### RNA sequencing analysis showed upregulation of genes involved in T cell signaling cascades

**Figure 3:**
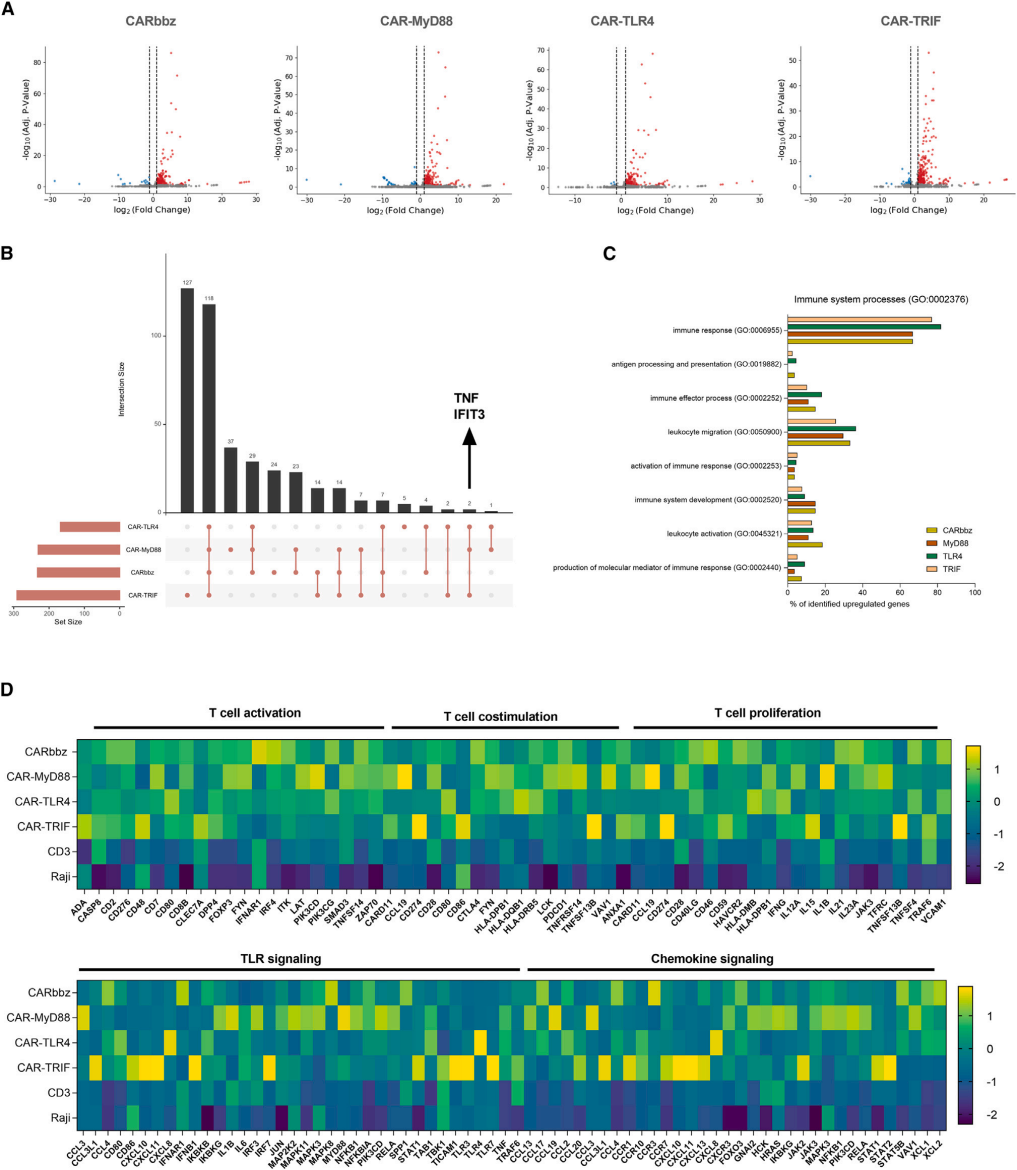
Transcriptome analysis of TLR4 signaling-based enhancement of CD19 CAR T cells following RNA-seq (A) Volcano plots (log_2_(fold change) vs. −log_10_(adjusted *p* value)) of differentially expressed genes of each CAR variant vs. untransduced cells. Significantly upregulated genes are colored red, and significantly downregulated genes are colored blue. The significance threshold to paint the data points was set to 0.1 (alpha), and horizontal lines show an absolute log_2_(fold change) threshold, set to 1, further painting significantly up/downregulated genes red and blue, respectively. In all four groups, GZMB and CSF2 are denoted as the top two significant points on the graph among the upregulated genes. (B) An overlap of significantly upregulated genes visualized by an UpSet plot showing the shared and unique genes among the four CAR groups. Filled circles represent the intersecting sets of genes, with bar plots showing the gene count per group and set. (C) Bar chart showing further annotated categories of immune system processes (GO: 0002376; level 1 PANTHER GO-Slim Biological Process). On the y axis are category names with GO accession numbers; percentage of identified upregulated genes represents several genes in the annotated category against the total number of genes in the immune system process category. Level 0 of the PANTHER GO-Slim Biological Process analysis is shown in Figure S3A. (D) Heatmaps of RNA-seq results showing upregulated and downregulated genes involved in specific immune system pathways in stimulated samples and both controls (Raji and nontransduced cells [“CD3”]) after 18 h. Colors represent the *Z* scores.

To gain further insight into the molecular mechanisms of our TLR4 signaling-derived CAR costimulation, we performed RNA sequencing (RNA-seq) of CAR T cells. This allowed us to compare the transcriptomes of our constructs with CARbbz. To provide an additional context, we included two additional controls, CD19-target Raji cells only and stimulated untransduced T cells (CD3+). DESeq2 analysis of selected CAR variants against stimulated untransduced cells identified several significantly upregulated genes (Figure 3A). The CAR-TRIF variant exhibited the highest number of upregulated genes as well as the largest set of distinct genes (Figures 3A and 3B). When comparing genes across all four CAR groups, we discovered a common subset of 118 genes (Figure 3B). Universal among the increased expression levels in all groups were molecules involved in T cell activation and the immune response, such as granzyme B (*GZMB*), *IFN-γ*, and colony-stimulating factor 2 (*CSF2*). GZMB and IFN-γ have antitumor effector functions, and upregulation of *CSF2* is highly prevalent in activated CAR T cells.^39^ TNF and interferon-induced protein with tetratricopeptide repeats 3 (*IFIT3*) were singled out for specific upregulation in the context of innate immunity costimulatory CARs (CAR-MyD88, CAR-TLR4, CAR-TRIF) in comparison to CARbbz (Figure 3B). Upregulation of the *TNF-α* gene can enhance T cell proliferation and survival, and TNF-α can modify the vasculature of solid tumors.^40^^,^^41^ While primarily associated with antiviral innate immunity, IFIT3 can also exert an indirect effect, leading to a decrease in cancer cell proliferation and the suppression of cancer.^42^

Next, we employed a PANTHER classification system^43^ to classify and annotate the list of upregulated genes into distinct Gene Ontology (GO) slim terms using the biological process annotation dataset (Figure S3A). The percentages of upregulated genes were similar between samples. We further focused on the genes in the immune system process category (GO: 0002376), annotating 7 “level 1” categories with most genes, set in the immune response category (GO: 0006955), followed by leukocyte migration category (GO: 0050900) (Figure 3C). To look deeper, we examined genes involved in T cell activation, T cell costimulation and proliferation, and genes involved in TLR signaling and chemokine signaling (Figure 3D). Aligned with expectations, the corresponding CAR groups exhibited significant and high expression of the *TICAM1* (*TRIF*), *TLR4*, or *MyD88* gene, providing compelling evidence of signaling modulation (Figure 3D). We observed the upregulation of genes that are involved in T cell activation and T cell stimulation, particularly in the CAR with MyD88 and TRIF domains. As anticipated from canonical TRIF signaling, CARs incorporating the TRIF domain exhibited extensively increased expression of genes involved in type I IFN signaling, while CARs incorporating the two other domains showed only a slight increase (Figure S3B), even though TLR4 signaling is upstream of both MyD88 and TRIF. Genes playing a crucial role in chemokine signaling were most substantially upregulated in the CAR-TRIF and CAR MyD88 groups. Some of the highly expressed chemokines likely promote T cell infiltration and could increase CAR T cell presence in the TME.^44^ The upregulation of these genes in CAR T cells can lead to increased T cell activation, proliferation, and effector functions, confirming our previous results for the higher immune response against tumors in comparison to CARbbz.

### Improved CAR T cell function in a solid-tumor model

**Figure 4:**
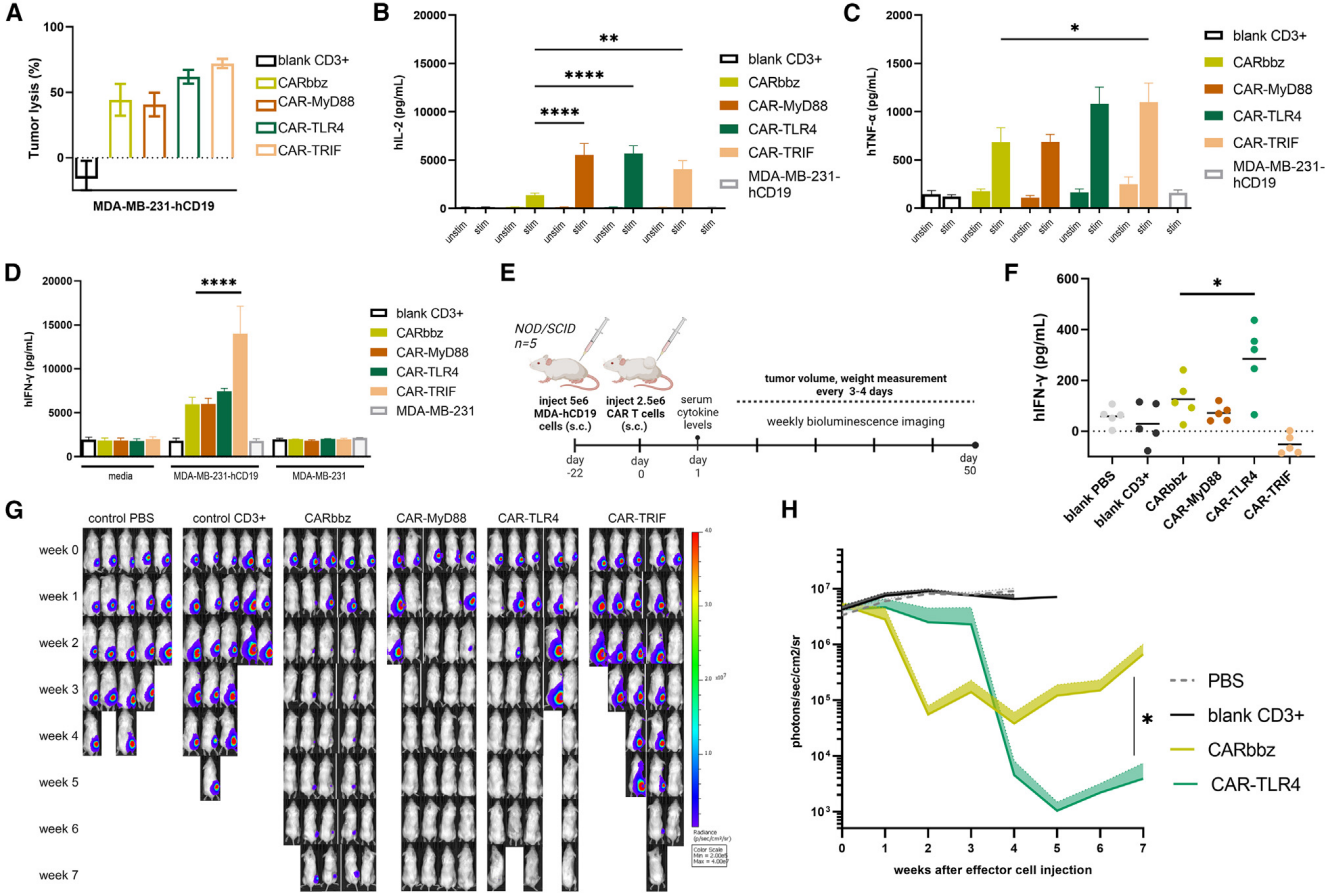
Augmented therapeutic efficiency of TLR4 signaling-domain-based CD19 CAR T cells in a solid-tumor model (A) MDA-MB-231-hCD19 tumor cell lysis after cocultivation at an E:T ratio of 10:1 at 24 or 48 h. Data are mean ± SEM from three different donors (*n* = 3). The concentrations of (B) hIL-2, (C) hTNF-α, and (D) IFN-γ were examined in the CD19-MDA breast cancer tumor cell line 48 h after coculture, maintaining a ratio of 10:1. Unstimulated samples with media only were measured as well. Significance in hTNF-α is shown between CARbbz and CAR-TRIF. IFN-γ secretion for unmodified breast cancer cell lines is also displayed. Data were collected from three different donors (*n* = 3). (E) Xenograft solid-tumor model was established by subcutaneous injection of 5 × 10^6^ MDA-MB-231-FLuc-hCD19 cells. The indicated CAR T cells at a dose of 2.5 × 10^6^ were injected peritumorally on day 22. Mice serum was collected 1 day after CAR T cell application. (F) hIFN-γ serum levels 1 day after treatment. Each dot represents the value of the respective mouse. (G) Bioluminescence images of MDA-hCD19-burdened mice on weeks 0–7 are depicted for each group. (H) Tumor average flux curves of the mice in each group are shown. Statistical difference in the seventh week was calculated between CARbbz and CAR-TLR4. Data were sourced from all mouse BLI images homogeneously scaled and BLI signal was measured with region of interest (ROI) analysis and with average background BLI subtraction. Data are mean ± SEM (*n* = 5); statistical differences were calculated by two-way ANOVA with Tukey’s multiple comparisons. ∗*p* < 0.05, ∗∗*p* < 0.01, ∗∗∗*p* < 0.001, ∗∗∗∗*p* < 0.0001.

Based on our findings in Raji cancer model, we explored whether this modification could contribute to resolving limitations in current CAR T cell therapies in solid-tumor treatments. To do so, we established a solid-cancer model based on the breast cancer cell line, given its prevalence as one of the most diagnosed cancers. We genetically modified the MDA-MB-231-FLuc tumor cell line to stably express hCD19 at the plasma membrane (Figure S4A). Next, we performed an *in vitro* killing assay that again confirmed increased cytotoxicity of CAR TLR4 and CAR-TRIF cells (Figure 4A). We also confirmed that these CARs did not nonspecifically kill breast cancer tumor cell lines lacking targeted antigens (Figure S4B). The secretion of cytokines, indicating increased CAR T cell activity and killing capacity, was significantly higher in some variants than in a standard CARbbz (Figures 4B–4D), and, additionally, we confirmed the absence of tonic signaling (Figure 4D). Surprisingly, *in vitro*, the results did not align with our initial expectation of a significant increase in hTNF-α expression in our CARs, as indicated by the RNA-seq data. Nonetheless, stimulated CAR-TRIF cells secreted significantly more hTNF-α than CARbbz cells (Figure 4C).

To determine the therapeutic efficacy of our TLR-based CAR T cells in solid tumors, we established a breast cancer xenograft solid-tumor model in NSG mice (Figure 4E), which lack their adaptive immune system.^45^ All mice developed palpable tumors, and, approximately 3 weeks later, the mice were grouped based on average tumor volume, measuring approximately 50 mm^3^. One day after CAR T cell application, we observed significantly higher values of hIFN-γ in the sera of the TLR4 group than in the sera of the CARbbz group (Figure 4F), confirming higher CAR T cell activation in a solid-cancer model. Control group tumors displayed exponential growth, while tumors treated with CARbbz responded rapidly. Within the MyD88 and TLR4 treatment groups, four mice exhibited a positive response to therapy, and one mouse from each group failed to respond. In contrast, the performance of the CAR-TRIF T cells *in vivo* was suboptimal, with a slow response and only two mice experiencing elimination of their solid tumors (Figures 4G, S4C, S4D, and S4F). To assess the long-term effects of the investigated CARs, we continued monitoring the animals for 50 days. Interestingly, we observed that complete eradication of tumor burden was not achieved with CARbbz treatment. The BLI signal in the CARbbz group exhibited a gradual increase after a few weeks but not in TLR-based CAR T cell-treated animals, suggesting tumor relapse in CARbbz-treated animals (Figures 4G, 4H, S4E, and S4F). Tumor relapse was not palpable; nevertheless, the MDA-MB-231 breast cancer cell line used in our study is known to exhibit a relatively slow proliferation rate,^46^ and BLI data allowed for the sensitive and earlier detection of tumor development.^47^ Given the potential occurrence of graft-versus-host disease (GVHD) at a later stage,^48^ we decided not to extend the experiment as it had already reached its upper threshold in duration. These data suggest that the incorporation of an additional TLR-costimulatory domain, such as MyD88 or TLR4, may contribute to the long-term remission and enhanced persistence of CAR T cells, in addition to the enhanced cytotoxicity. During the final week of observation, one mouse from the CARbbz group and one from the CAR TLR4 group were excluded from the study due to symptoms resembling GVHD.^48^ Within the TRIF group, one mouse experienced a loss exceeding 15% of its initial body mass within the sixth week post treatment and was withdrawn from the experiment. The remaining mice did not exhibit drastic weight loss, as depicted in Figure S4G. After 50 days, we ended the experiment, gathered spleens from the residual mice, and extracted the splenocytes. We checked for the expression of hCD3 on the surface of splenocytes. Mice treated with TLR CARs exhibited a considerably higher proportion of hCD3+ cells in their spleens relative to mice that received the CARbbz variant (Figure S4H). These findings further indicate the notion of prolonged persistence of TLR CARs in comparison to the standard CARbbz.

## Discussion

Here, we show that the incorporation of costimulatory domains derived from TLR4 signaling pathways leads to elevated therapeutic CAR T cell cytotoxicity, cell activation, and possibly CD19 CAR sustainability. These findings were also shown in a solid-cancer model *in vivo*, where we observed higher human CD3 cell proliferation in spleen and higher sera hIFN-γ levels. Furthermore, we noted long-term remission with CAR-TLR4 and CAR-MyD88 T cell therapy compared to CARbbz. Although some advantages of CAR-TLR4, such as higher cytokine level, were seen in hematological cancer model, there was no increase in survival, perhaps due to the upregulation of PD-1 and LAG-3 exhaustion markers observed in CAR T cells, before administering them in mice provoking lower CAR T cell persistence and immunotherapeutic function.^49^^,^^50^^,^^51^ It was shown previously that combining the MyD88 costimulatory domain with CD40 and tethering it to a CAR results in enhanced antitumor activity as well as improved CAR cell survival and proliferation.^19^^,^^20^^,^^21^^,^^52^^,^^53^ The main limitation of that design lies in the observed tonic activity of the construct with MyD88 fused to the CAR domain in addition to CD40, which we did not observe in any of our TLR-based CAR T constructs, where TLR signaling domains are expressed on a separate polypeptide chain. The absence of tonic activity is important, as it may lead to T cell exhaustion, limiting immunotherapeutic efficiency or decreasing persistence.^54^ In another study, incorporation of TLR2 TIR signaling domains into a third-generation CD19 CAR comprising CD28 and CD3ζ exhibited enhanced expansion, persistence, and effector function both *in vitro* and *in vivo*,^23^^,^^55^ demonstrating the significance of incorporating TLR signaling domains to enhance the killing efficiency of CAR T cells.

Cells triggered to release type I IFNs possess the ability to modulate various aspects of tumor cell behavior, including growth, proliferation, differentiation, migration, and apoptosis, through their cytotoxic, cytostatic, and antiangiogenic properties.^56^^,^^57^ On the other hand, prolonged exposure to type I interferons may contribute to immune dysfunction.^58^ Recently, CAR T cells were engineered to release type I IFNs, and antitumor activity decreased in subsequent rounds of stimulation with tumor cells.^59^ CAR T cell loss and upregulation of the inhibitory receptors due to type I IFN-induced signaling by oncolytic viruses were also observed in a separate study.^60^ Our study also demonstrated that the incorporation of the TRIF activation domain, which triggers the release of type I IFNs, into CAR T cells as a costimulatory domain could result in CAR T cells with impaired functionality *in vivo*.

When activated, CAR T cells produce IL-2, a crucial factor for their survival and function. The elevation in IL-2 secretion could potentially enhance the survival of infused CAR T cells, thereby improving long-term outcomes,^61^ as we also observed here in the TLR4 domain incorporated into CAR T cells. IL-2 has also been exploited therapeutically as an immunostimulatory agent capable of inducing remission in metastatic renal cell carcinoma and melanoma.^62^ Despite its effectiveness, it was implicated in producing dose-limiting toxicities, resulting in severe adverse effects. In our experiments, we observed an increase in hIL-2 secretion *in vitro* but found no toxicities *in vivo*.

CAR-MyD88 and CAR-TLR4 tested in this study appeared to display a somewhat slower response in solid tumors *in vivo*, yet they accomplished full remission in all responding subjects for the duration of the experiment, a level of success that CARbbz was unable to reach. This outcome could be potentially linked to the activation of TLR signaling pathways, resulting in enhanced T cell proliferation, as shown by the increased proportions of CD3+ cells within the spleen in our study. Prinzing et al. also reported similar results, where they also observed increased persistence in CAR T cells by incorporating CD40/MyD88.^19^ Foster et al. introduced chemical regulation of the MyD88/CD40 domain to limit constitutive activity and potentiate CD3 stimulation-mediated T cell activation.^20^ In the constructs presented here, tonic activity was absent; therefore, there was no need for additional chemical regulation, although it is possible that chemical crosslinking might additionally potentiate activation.

While a potential downside of higher activation could manifest in the elevated CRS, the *ex vivo* experiments, combining relevant immune cells and *in vivo* results, did not demonstrate statistically significant increases in hIL-6 levels in TLR CARs, which we considered an important observation regarding their safety. It should be noted, though, that NSG animal models present some limitations, as they lack several crucial functional cells that could play a major role in CRS pathogenesis.^63^ Further experimentation involving immunocompetent mice models would be appropriate to comprehensively investigate these findings. On the other hand, clinical trials concerning other TLR-based CAR T cells reported no indication of an elevated CRS risk,^23^ which is a major factor for the wider adoption of CAR T cell therapy.

In addition to T cells, the CARs presented here may also be functional in natural killer (NK) cells or particularly macrophages, where engagement of the TLR signaling pathways is likely to trigger M1 polarization, as demonstrated recently by Duan et al., where the intracellular TLR4 signaling domain was included.^64^ Similarly, Lei et al. recently demonstrated that the incorporation of the TLR4 TIR domain in second-generation CARs within induced pluripotent stem cell-derived macrophages (iMACs) led to markedly enhanced cancer killing. Their research highlighted that CARs with TIR domain not only promote the engulfment of apoptotic tumor bodies but also drive M1 polarization through the NF-κB signaling pathway.^65^ This suggests that TLR4 bears important hallmarks for enhanced therapeutic effects beyond T cells. The benefits of incorporating TLR domains into NK cells were also already assessed. When combined with inducible MyD88/CD40, NK CAR cells exhibited enhanced proliferation and augmented cytotoxicity.^53^

In conclusion, this study demonstrates that the incorporation of TLR4 signaling costimulatory domains can modulate CAR T cell activation. Remarkably, the truncated TLR4 variant expressed as a separate membrane-anchored polypeptide chain^30^ lacking tonic activity and exhibiting moderately enhanced immunotherapeutic properties presents a novel CAR design candidate for cancer therapy.

## Material and methods

### Cell lines

Human embryonic kidney (HEK) 293T and human Jurkat cells were purchased from American Type Culture Collection (ATCC). Raji cells were a kind gift from N. Kopitar-Jerala, and BCWM-FLuc cells were a kind gift from S.P. Treon.^66^ K562-FLuc cells were a kind gift from S. Wälchli.^67^ The parental MDA-MB-231-FLuc cell line, a kind gift from T. Petan, was modified with a CD19-expressing lentiviral vector to express human CD19 as described elsewhere.^68^ HEK293T and MDA-MB-231 cells were cultured in DMEM (Invitrogen Life Technologies) supplemented with 10% (v/v) heat-inactivated fetal bovine serum (FBS) (Invitrogen Life Technologies), whereas Jurkat, BCWM, and Raji cells were grown in RPMI1640 (Invitrogen Life Technologies) medium supplemented with 10% (v/v) heat-inactivated FBS (Invitrogen Life Technologies). Cell lines were kept in culture for up to 20 passages. Cell lines were maintained at 37°C in 5% CO_2_.

### Plasmids

Plasmids that express human MyD88 and human TLR4 were a kind gift from T. Espevik, MAL- and TRIF-expressing plasmids were synthesized, and myr-MyD88 was designed as previously described.^24^ Plasmids designed for this study were constructed through standard procedures of molecular cloning or the Gibson assembly method,^69^ and various linkers were inserted as part of PCR amplification primers along with overlapping sequences for Gibson assembly. CD19 CARbbz was constructed as previously described.^68^ For experiments in Jurkat cells, plasmids in the pcDNA3.1 (Invitrogen) backbone were used. Selected constructs were recloned and inserted into a third-generation lentiviral expression vector, pLVX-Puro (Addgene, 141395) under the regulation of a human EF-1α promoter. Additionally, pVSV-G (Addgene, 138479) and psPAX2 (Addgene, 12260) were used as packaging plasmids for lentivirus production. All constructs were verified with DNA sequencing.

### Cell electroporation

Jurkat cells (3 × 10^7^ cells/mL) were electroporated in 100 μL of electroporation tips by a Neon electroporation system (Thermo Fisher Scientific) using R buffer with adjusted parameters to 1,600 V voltage, 10-ms pulse, and three pulses. The total amount of DNA per electroporation was 10 μg.

### Isolation and expansion of human T cells

Human T cells were obtained from healthy donors and handled following ethical and safety procedures. Peripheral blood mononuclear cells (PBMCs) were isolated from the buffy coat of healthy donors by Lymphoprep (Stemcell) density gradient medium centrifugation. Primary human T cells were isolated by negative magnetic selection (Pan T Cell Isolation Kit, Miltenyi Biotec) according to the manufacturer’s instructions. CD3+ cells were maintained in RPMI medium (Invitrogen Life Technologies) supplemented with 10% heat-inactivated (v/v) FBS (Invitrogen Life Technologies), 25 μL/mL of ImmunoCult Human CD3/CD28 T cell activator (Stemcell Technologies), and 100–200 U/mL of human recombinant IL-2 (StemCell Technologies) for 48 h before viral transduction.

### Lentiviral production and generation of gene-modified T cells

Lentiviral particles were generated through the transient transfection of plasmids into the HEK293T cell line as previously described.^68^ Concentrated lentivirus particles were resuspended in PBS and stored at −80°C. Titers were determined by transducing 1 × 10^5^ Jurkat cells in duplicate with serial dilutions. Two days later, the cells were harvested and washed twice with PBS, and CAR expression based on surface Myc staining on the surface of Jurkat cells was determined by flow cytometry. Myc tag was fused to all CAR constructs at the N terminus to allow for the detection of CAR expression by the anti-Myc antibody. Titers were determined based on the percentage of CAR-positive Jurkat cells and expressed as transducing units/mL (TU/mL).

Human T cells were obtained from healthy donors. Samples were obtained and handled following ethical and safety procedures. The study protocol was approved by the National Medical Ethics Committee of the Republic of Slovenia (0120-21/2020/4 and 0120-204/2023/6). Primary T cells were transduced with CAR lentiviral particles at a multiplicity of infection (MOI) of 5 and expanded for 7–10 days in G-Rex six-well plates (Wilson Wolf) with medium change and 100 U/mL IL-2 (StemCell Technologies) replacement every 2–3 days. For controls, untransduced T cells from corresponding donors were grown under the same conditions. CAR T cells were assessed for transduction efficiency via flow cytometry by measuring Myc-tag expression on the surface. For all functional assays, CAR T cells were normalized for transduction efficiency using untransduced cells from the same donor that were grown at the same time. Multiple donors were used.

### Flow cytometry

To determine the phenotype of the CD19 CAR T cells, fluorescein isothiocyanate (FITC) anti-human CD62L (BioLegend), Pacific Blue anti-human CD45RA antibody (BioLegend), human CD8-FITC (BW135/80, Miltenyi Biotec), human CD4-VioBlue (Miltenyi Biotec), anti-human PD1-PE-Vio770 (Miltenyi Biotec), Brilliant Violet 605 anti-human LAG-3 (BioLegend), anti-human TIM-3-APC-Cy7 (BioLegend), anti-human CD3 VioGreen (Miltenyi Biotec), and Myc-Tag (9B11) Mouse monoclonal antibody (mAb) Alexa Fluor 647 Conjugate or Alexa Fluor 488 Conjugate (Cell Signaling Technology) were used. For staining splenocytes, we used anti-human CD3 VioGreen (Miltenyi Biotec). All staining was performed for 30 min at 4°C in the dark. Cells were washed with fluorescence-activated cell sorting (FACS) buffer (PBS, 2% FBS) and resuspended in 0.2 mL of FACS buffer. The determination of viable cells was performed using 7-AAD Viability Staining Solution (Thermo Fisher Scientific). Flow cytometry was performed on a Cytek Aurora Flow Cytometry System (Cytek Biosciences) with SpectroFlo software, and gating strategies are shown in Figure S5. FMO controls were included in relevant experiments. Spectral unmixing with single stain controls was performed.

### Cytotoxicity assays

CAR and T cells were washed to remove hIL-2 and resuspended in RPMI medium. For the *in vitro* functional studies, cell lines (Raji-FLuc, BCWM-FLuc, MDA-MB-231-FLuc, MDA-MB-231-FLuc-hCD19) were used to assess CD19 CAR T-mediated cell killing at the indicated effector-to-target (E:T) ratios for the indicated time points. After a designated period, D-luciferin (Xenogen) was added to the cocultured cells to a final concentration of 150 μg/mL. BLI signal was measured using an IVIS Lumina Series III (PerkinElmer). Data were analyzed with Living Image 4.5.2 (PerkinElmer). The percentage of specific lysis was calculated from average radiance values (ARV) using the following formula: % specific lysis = 100 × (spontaneous death ARV − test ARV)/(spontaneous death ARV − background ARV).

### Cytokine production assay

Plates used for cytotoxicity were spun at 1,500 rpm for 5 min to pellet the cells, and culture supernatants were collected. Cytokine concentrations in the supernatants were measured using hIL-2 ELISA, hIFN-γ ELISA, hTNF-α, hIL-6, and mIL-6 ELISA (Thermo Fisher Scientific) kits according to the manufacturer’s instructions. Washing between the incubation steps was performed using a HydroSpeed plate washer (Tecan). A microplate reader SynergyMx (BioTek) was used to measure endpoint absorbance.

### Bulk RNA-seq sample preparation

CAR T cells and tumor cells were incubated at a 2:1 E:T cell ratio in duplicate. Total RNA was extracted after 18 h with the High Pure RNA Isolation Kit (Roche) according to the manufacturer’s recommendation and later purified and concentrated with the RNA Cleanup Kit (Monarch). The RNA quantity and purity (A260/A280) ratio were measured on a Nanodrop spectrophotometer (Thermo Fisher Scientific), and the RNA integrity number (RIN) was determined using a 2100 Bioanalyzer with an Agilent RNA 6000 Pico kit (Agilent). Sample quality control, library preparation, rRNA, and globin depletion were performed by Azenta Life Sciences. Library preparation was performed according to their standard protocols and sequenced on Illumina NovaSeq paired-end 150-bp sequencing, 10 million read pairs.

### Bulk RNA-seq analysis

For RNA-seq analysis, we employed various tools within the RNAlysis software.^70^ Initially, we conducted paired-end Illumina TrueSeq adapter trimming using CutAdapt^71^ followed by Kallisto pseudoalignment to the human genome. The transcriptome index file and gtf file, derived from Ensembl v96 transctiptomes,^72^ were downloaded before analysis from the Kallisto transcriptome indices data source, and Kallisto was thus utilized to quantify gene expression across all samples.^73^

Differential expression analysis was performed using the R package DESeq2^74^ to compare a total of 35,606 gene transcripts between stimulated CAR samples (CARbbz, CAR-MyD88, CAR-TLR4, CAR-TRIF) and stimulated untransduced T cells (sample CD3). The resulting data were filtered by statistical significance (α = 0.1) and split by log_2_ (fold change) directions. Volcano plots were used for visualization. To annotate and categorize the up- and downregulated genes obtained from DESeq2, we used the PANTHER database online tool,^43^ focusing specifically on GO terms within the biological process subset.^75^^,^^76^ Results are visualized using bar plots. To identify and visualize the overlapping sets of upregulated genes among the four different experimental CAR groups, we utilized the UpSetR package in RStudio. To assess gene expression within specific immune pathways (T cell activation, costimulation, proliferation, TLR signaling, and type I interferon signaling), we first normalized the read counts generated by Kallisto pseudoalignment using the relative log expression method. Genes with expression levels below the 0.5 threshold across all samples were excluded. Subsequently, *Z* scores were calculated to compare the expression of respective genes between all six sequenced groups. Results are visualized using heatmaps with a viridis color map.

### *In vivo* mouse experiments

NOD. CB17-Prkdc^scid^ IL2rgt^m1^/BcgenHsd mice were purchased from Envigo, Italy, and bred under pathogen-free conditions. Mice were maintained in a 12-/12-h dark/light cycle at 40%–60% relative humidity and a room temperature of 20°C–24°C. Laboratory animals were housed in IVC cages (Techniplast) and fed standard chow (Mucedola), and tap water was provided *ad libitum*. All animals used in the study were healthy and accompanied by a health certificate from the animal vendor. Health/microbiological status was confirmed by the FELASA-recommended Mouse Vivum panel (QM Diagnostics). Animal experiments were performed according to the EU Directive 2010/63/EU and were approved by the Administration of the Republic of Slovenia for Food Safety, Veterinary and Plant Protection of the Ministry of Agriculture, Forestry and Foods, Republic of Slovenia (permit numbers U34401-28/2019/20, U33401-18/2023/1).

For xenograft cancer studies, 8- to 12-week-old males or females were selected. A xenograft lymphoma model was created by injecting 1 × 10^6^ Raji cells intraperitoneally. On day 5, the indicated CAR T cells were injected intraperitoneally at a dose of 5 × 10^6^. The tumor BLI signal was monitored weekly. The mice were given 150 mg/kg D-luciferin (Sigma) based on their body weight and were anesthetized using isoflurane inhalation. After 10 min, the animals were subjected to *in vivo* imaging using IVIS Lumina Series III (PerkinElmer). The data obtained were analyzed using Living Image 4.5.2 (PerkinElmer) with background subtraction. After a defined period, the mice were humanely sacrificed.

A solid xenograft tumor model was established by subcutaneously injecting 5 × 10^6^ MDA-hCD19 cells into 8- to 12-week-old female mice. Body weight and tumor size were measured via calipers every 3–4 days. Tumor burden was calculated using the formula V = (a × b × c × Π)/6. When tumors were approximately 50 mm^3^ in volume, PBS, untransduced T cells, or CAR T cells were administered peritumorally at 2.5 × 10^6^/mouse. The tumor BLI signal was measured on a weekly basis. The mice received 150 mg/kg of body weight of D-luciferin (Sigma) and were anesthetized by isoflurane inhalation anesthesia. After 10 min, the animals were imaged *in vivo* with IVIS Lumina Series III (PerkinElmer). Data were analyzed with Living Image 4.5.2 (PerkinElmer) with background subtraction. Blood was collected from mice at the indicated times by tail-clip bleeding. Following clotting, blood was centrifuged at 3,000 rpm for 20 min at 4°C. Serum was immediately stored at −80°C until analysis. Animals were humanely euthanized when a certain dimension of the observed tumor reached 12 mm, the tumor ulcerated, or the animals lost over 20% of their initial body mass. Single-cell suspensions from spleens without enzymatic treatment were prepared by using the tissue dissociator gentleMACS Dissociator according to the manufacturer’s instructions (Miltenyi Biotec).

### Statistical analyses

FlowJo v10.8.1 software (BD Life Sciences) was used to analyze the data obtained from the flow cytometry experiments. Graphs and statistical analyses were prepared with GraphPad Prism Software (GraphPad Software). Data are presented as the means ± SEMs. One-way or two-way ANOVA followed by Tukey’s multiple comparisons test was used to statistically compare data. Significance was considered for *p* < 0.05 as follows: ∗*p* < 0.05, ∗∗*p* < 0.01, ∗∗∗*p* < 0.001, and ∗∗∗∗*p* < 0.0001.

## Data and code availability

The authors confirm that the data supporting the findings of this study are present within the article and its supplemental figures. All other data are available from the authors of the paper upon request.

## Supplemental information

Document S1. Figures S1–S5

Document S2. Article plus supplemental information
