# Supplementary material for: Toll-like receptor 4 signaling activation domains promote CAR T cell function against solid tumors
Source: Mol Ther Oncol. 2024 May 14;32(2):200815. doi: 10.1016/j.omton.2024.200815 (PMC11152746; doi:10.1016/j.omton.2024.200815)
Supplement: Document S1. Figures S1–S5 [file mmc1.pdf]

OMTON, Volume *32*

Supplemental information

Toll-like receptor 4 signaling activation domains promote CAR T cell function against solid tumors

Veronika Mikolič, Jelica Pantović-Žalig, Špela Malenšek, Matjaž Sever, Duško Lainščček, and Roman Jerala

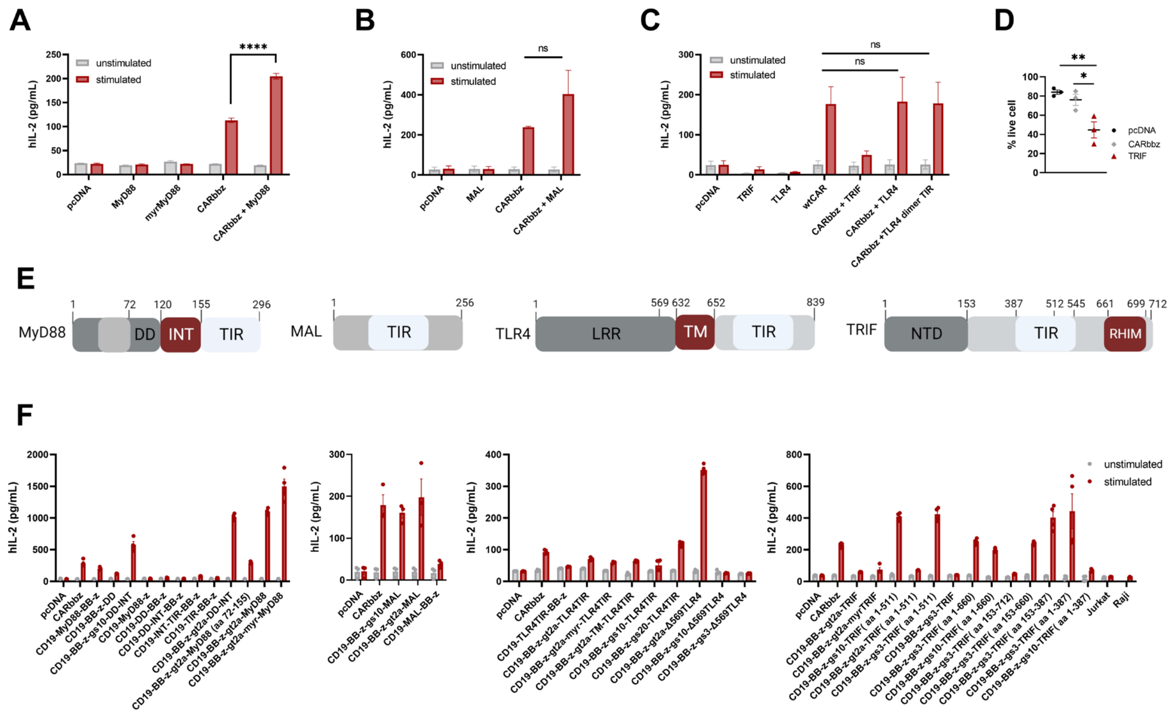

Figure S1: Validation of plasmid-expressed TLR-activation domains in Jurkat cells. (A) Cytokine production from electroporated Jurkat cells with CARbbz and MyD88-expressing plasmids 48h after co-cultivation with Raji cells with effector: target ratio of 10:1 (n=3). **(B)** IL-2 secretion from Jurkat cells that were electroporated with the CARbbz and MAL-expressing plasmid, following a 48 hours cocultivation with Raji cells (E:T=10:1), determined by ELISA (n=3). **(C)** IL-2 release from electroporated Jurkat cells with CARbbz, TRIF- and TLR4-expressing plasmids with subsequent 48h long co-cultivation with target CD19+ Raji cells (effector: target ratio of 10:1) (n=3). **(D)** Jurkat cells viability was assessed 24 hours after electroporation with depicted plasmids using Trypan blue staining (n=3). **(E)** Schematic illustration of tested TLR signaling components with annotated amino acid sequence intrinsic to their structural and functional motifs.[17, 24, 27] **(F)** Cytokine production from electroporated Jurkat cells with CD19 CAR constructs following 48h cocultivation with target Raji cells (effector: target ratio of 10:1). Data are mean ± SEM. Statistical differences are calculated by

one-way or two-way ANOVA with Tukey's multiple comparisons. *, P < 0.05; **, P < 0.01; ****, P < 0.0001.

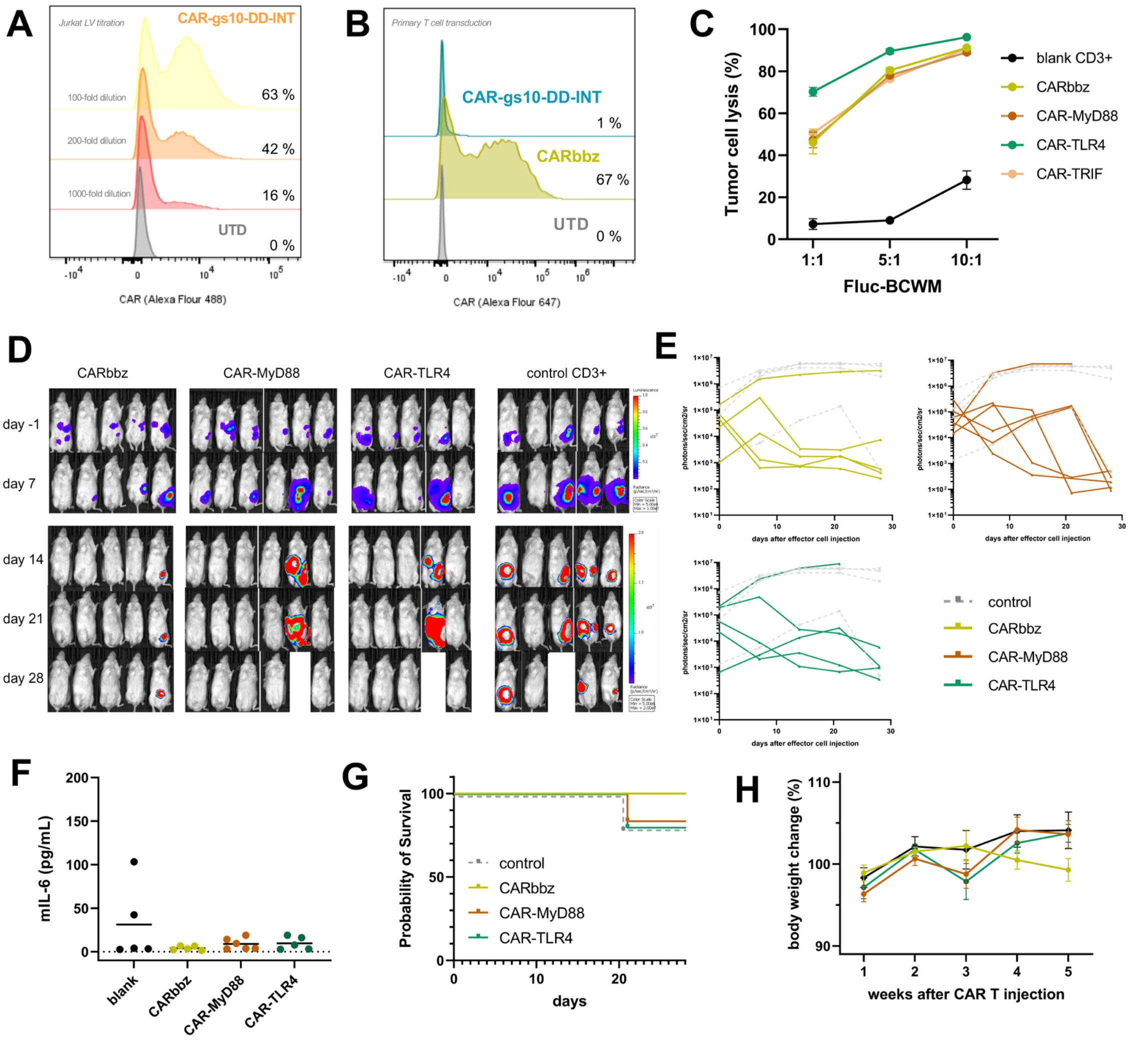

Figure S2: CAR-DDINT surface expression and in vivo effect of human TLR-based CD19 CAR T cells. (A) Histogram of surface expression of CAR-gs10-DD-INT following lentivirus titration efficiency on Jurkat cells with various dilution factors, as determined by flow analysis. Histograms displayed are representative data. **(B)** Comparison of virally transduced CD19 CAR surface expression on human CD3+ T cells for CAR-gs10-DD-INT, CARbbz and untransduced T cells. Representative examples are shown. **(C)** Cytotoxic effects of targeted killing on the CD19+ BCWM-FLuc cell line and CD19 CARs in vitro are demonstrated at the

indicated E:T ratio. The results displayed are three biological repeats collected from a single donor. Data are represented as mean ±SEM**.** **(D)** Bioluminescence imaging of NSG mice at days -1, 7, 14, 21 and 28 are depicted for each group, demonstrating the functional immunotherapeutic effect of administered CD19 CAR T cells. Schematic illustration of in vivo experiment, including timeline, administration paths and doses are displayed in Figure 2J. **(E)** Spaghetti plots demonstrate BLI signal for the individual mouse. (n=5-6). Data was sourced from all mice BLI images homogeneously scaled and with background BLI subtraction. **(F)** mIL-6 concentrations in mice serum 3 days after CAR T cell application. Each dot represents a single mouse (n=5-6). **(G)** The survival rates of mice were depicted using the Kaplan-Meier method. Survival curves were analyzed with the Log-rank test. **(H)** Change in body mass (depicted in % from the initial measurement) over a period of weeks.

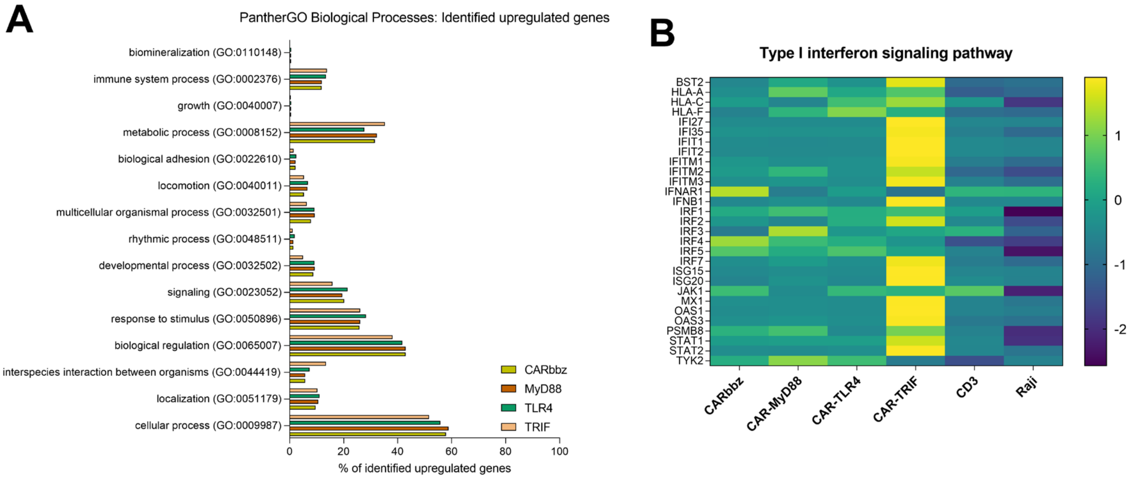

Figure S3: Transcriptome analysis following RNA-seq. **(A)** Bar chart displaying percentage of identified upregulated genes in biological processes (Level 0 of PANTHER GO-Slim Biological Process analysis). On the y-axis are category names with GO accession numbers, on the x-axis % of identified upregulated genes represent number of genes in the annotated category against total number. **(B)** Heatmap of RNA sequencing results showing upregulated

and downregulated genes involved in type I interferon signaling pathways in stimulated samples after 18h. Colors represent the z score.

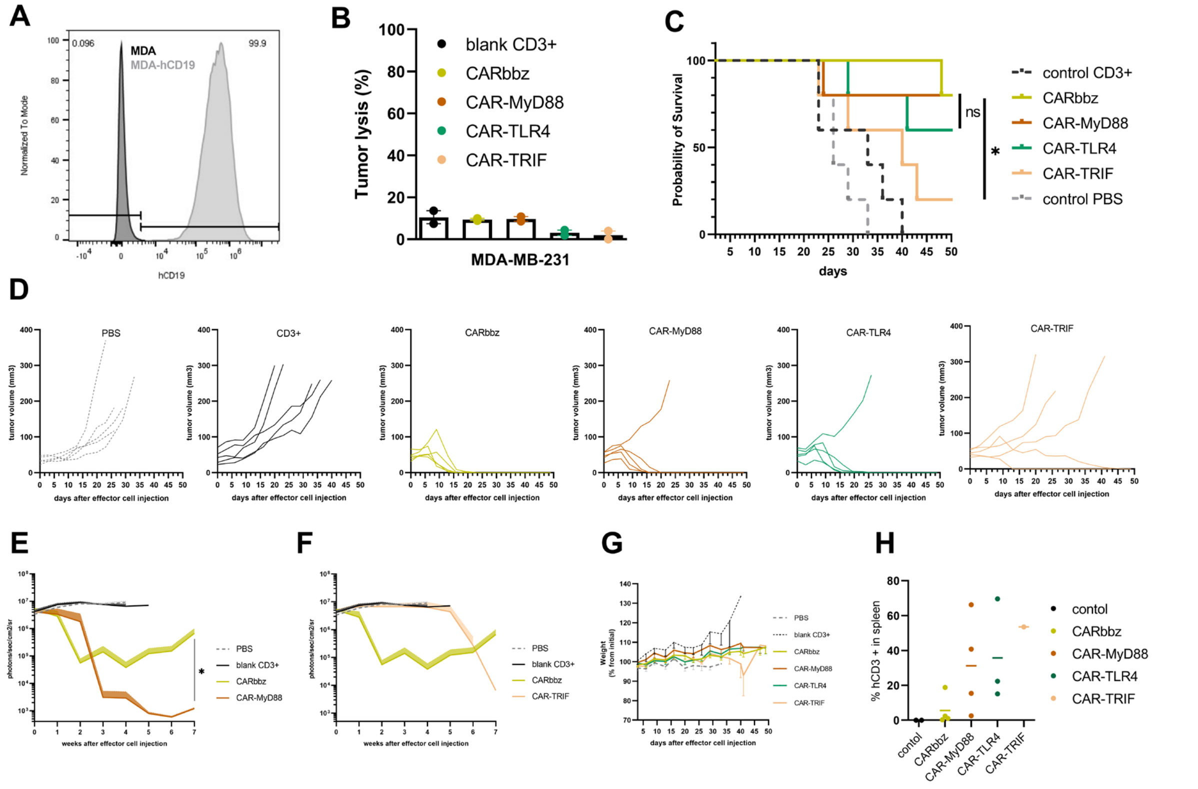

Figure S4: TLR-based CD19 CAR T cell effect on MDA-MB-231-CD19 solid tumor cell line model. (A) Histogram demonstrates surface expression of hCD19 on the genetically modified MDA-231-MB breast cancer cell line. **(B)** MDA-MB-231 which lacks targeted CD19 surface marker, demonstrated no unspecific CAR targeting as evidenced by the absence of tumor cell lysis 24h after cocultivation at E:T ratio 10:1. Data are mean ±SEM (n=2). **(C)** Kaplan Meier survival curve of mice (n=5). Survival curves were analyzed with the Log-rank test. Statistical difference shown between CARbbz and CAR-TRIF and no statistical difference between CARbbz, CAR-MyD88, and CAR-TLR4. Schematic illustrations of in vivo experiment, including timeline, administration paths, and doses are displayed in Figure 4E**. (D)** Tumor volume growth curves of the individual mice in each group are shown. Tumor average

BLI curves of the mice in CAR-MyD88 **(E)** and CAR-TRIF **(F)** groups with controls are shown. Data were sourced from all mice BLI images homogeneously scaled and with background BLI subtraction. Data are mean ± SEM (n=5). Statistical differences in the seventh week were calculated by two-way ANOVA with Tukey's multiple comparisons. **(G)** Changes in body weight relative to initial measurements on specific days following treatment administration. Data are mean ±SEM (n=5). **(H)** Mice spleens were collected at the end of the experiment, and single-cell suspensions were prepared. Subsequently, flow cytometry analysis was performed to determine the displayed percentages of hCD3+ cells in the spleens 50 days after CAR T cell administration. Lines on the graph represent mean values. (n=1-4) *, $P < 0.05$

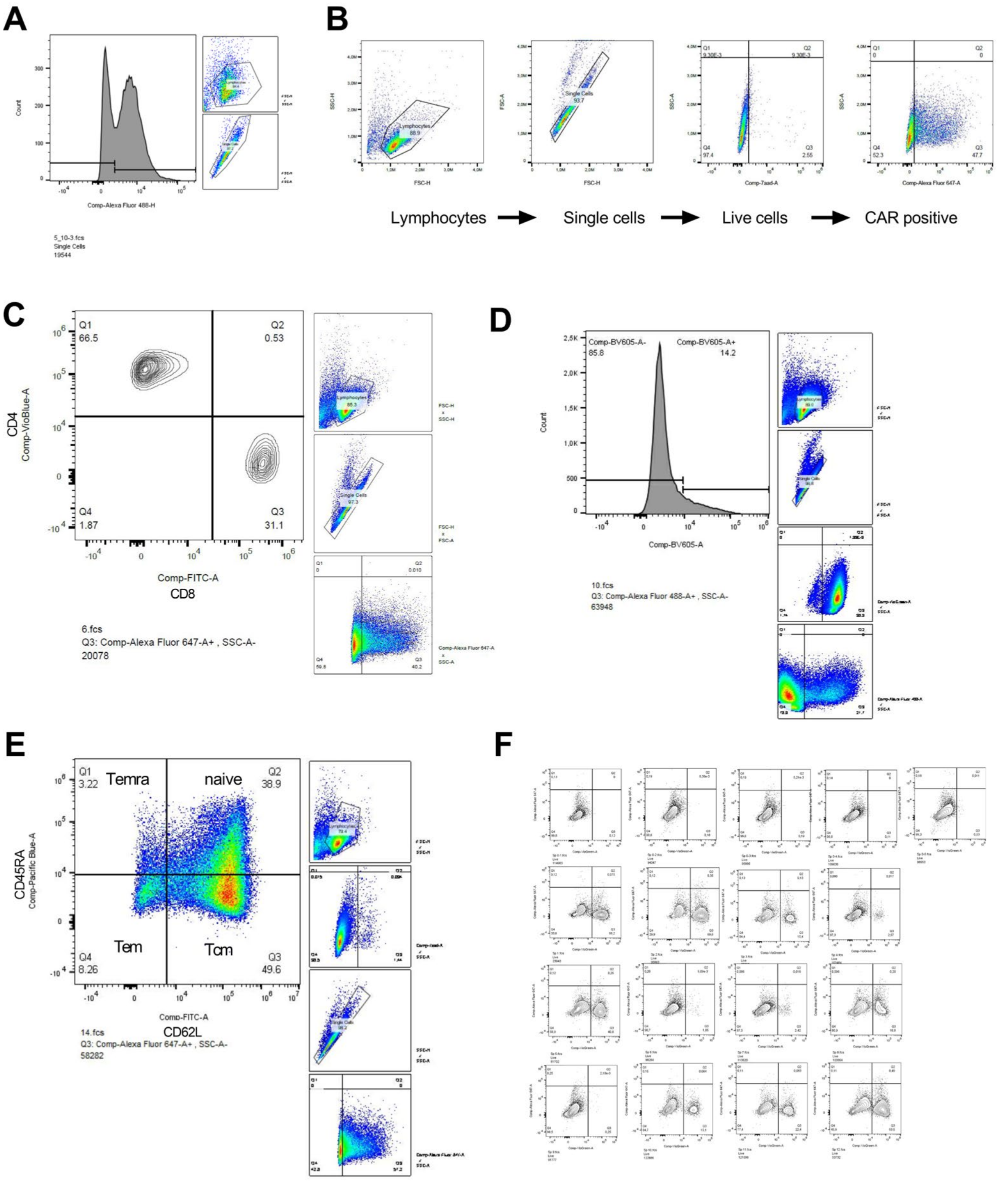

Figure S5: Flow cytometry gating strategies. (A) Backgating to identify CAR positive cells in a lentiviral titration experiment conducted on Jurkat cells (Figure S2A). **(B)** Gating methods for the identification of CAR-expressing cells one week following the transduction of primary human CD3 cells. **(C)** Ancestry gating to evaluate CD4/CD8 ratio phenotype of CAR T cells. **(D)** Example of gating approach for phenotyping exhaustion markers in CAR T cells. **(E)** Gating strategy to identify memory status of CAR T cells. **(F)** Provided graphs display flow cytometry results and the gating approach we used to analyze splenocytes for the expression of hCD3.
